# A Comparison of Phenolic Monomers Produced from Different Types of Lignin by Phosphotungstic Acid Catalysts

**DOI:** 10.1002/open.201900088

**Published:** 2019-05-23

**Authors:** Boyu Du, Bingyang Liu, Xing Wang, Jinghui Zhou

**Affiliations:** ^1^ Department Liaoning Key Laboratory of Pulp and Papermaking Engineering Dalian Polytechnic University Dalian Liaoning 116034 China; ^2^ Guangxi Key Laboratory of Clean Pulp & Papermaking and Pollution Control College of Light Industry and Food Engineering Guangxi University Nanning 530004 China; ^3^ State Key Laboratory of Pulp and Paper Engineering South China University of Technology Guangzhou 510640 China

**Keywords:** lignin, depolymerization, renewable resources, catalysts, phenolic monomers

## Abstract

Herein we studied the chemical structure of different types of lignin samples and the potential to prepare phenolic monomers was illustrated by phosphotungstic acid catalysts. Different types of H/G/S lignin components had different structures. The lignin extracted from poplar had the highest molecular weight and β‐O‐4 aryl ether contents, followed by pine and straw lignin samples. After depolymerization by PTA catalyst, the yields of phenolic monomers detected was 8.06 wt % (poplar), 5.44 wt % (pine) and 4.52 wt % (straw), respectively. Further, the ratios of H/G/S in the phenol monomers were also different, indicating that the S, G and H types structural units were continuously transformed with each other during the reaction. In our study, the change in the types of lignin samples resulted into an improvement of the distribution of phenolic products, and also the selectivity of phenolic monomers significantly.

## 1 Introduction

With the development of industry and growth of the world population, the global consumption of fossil fuels and related environmental problems have steadily increased. The search for sustainable resources used for new energy sources and materials is still ongoing.^1^ Lignin is a relatively abundant renewable resource currently composed of a variety type of aromatic hydrocarbon compounds.^2^ It is the only big‐capacity reproducible material which includes an aromatic skeleton and its exhibits various attractive features, such as a largest renewable source for aromatics, high carbon content, high thermal stability and favorable stiffness.^3^ According to the aromatic feature of lignin, recent advances have demonstrated the potential for the conversion of lignin to a spectrum of aromatic compounds via catalysis, thereby replacing the traditional way from fossil resources.^1^, ^2^,^4^ Lignin is mainly an amorphous tridimensional polymer of syringyl (S), guaiacyl (G), and p‐hydroxyphenyl (H) units, and some non canonical subunits.^4^ We consider that the structure of lignin is extremely different because of different types of lignin samples. For example, softwood samples contain more guaiacyl units, and softwood samples contain a mix of guaiacyl and syringyl, while grass samples have a mixture of these three aromatic units.^5^ Due to the complex structure of different types of lignin samples, this structure may be a key factor affecting the yield and distribution of phenolic monomers products in the later reactions of lignin degradation.^6^

Chemical disassembly of lignin using non‐noble metals is one of the most efficient approaches to obtain mono phenols from lignin. Guo et al. studied that a remarkably effective method for the chemoselective cleavage of the C−O bonds of typical β‐O‐4 model compounds and the deconstruction of lignin feedstock was developed by using tungsten carbide as the catalyst.^7^ High yields of C−O cleavage products (up to 96.8 %) from model compounds and liquid oils (up to 70.7 %) from lignin feedstock were obtained under low hydrogen pressure (0.69 MPa) in methanol. Yang et al. proposed a high‐efficient heterogeneous acid H‐ZSM‐5 catalysts depolymerization process with highly controllable products.^8^ Besides, Deng et al. reported that Keggin‐type Cs^+^ salts of polyoxometalates, showed firm acidity and catalyze the conversion of lignin to phenolic monomers.^9^ Du et al. studied that the use of phosphovanado molybdate for the cleavage of oxidative C−C bonds.^10^ Building on the above results, we discovered that polyoxometalate exhibits similar catalytic performance to those in a variety of reactions involving solid acids. Nevertheless, the impact of different types lignin on the conversion performance of phosphotungstic acid (PTA) polyoxometalate is unclear yet.

In the present study, three types of lignin samples extracted by ethanol solvent were analyzed and used for the depolymerization of the PTA polyoxometalates catalysts in ethanol/water. Then, we explored characterization of lignin samples from different types of samples (poplar, pine and straw) and tried to connect the yields and distribution of phenolic monomers with the structure of lignin samples. The final goal was decided to the influence of the different types of lignin structure on the yield and distribution of phenolic monomers.

## 2 Results and Discussion

### 2.1 2D‐HSQC NMR of the Different Types of Lignin Samples

Chemical analysis with the 2D‐HSQC NMR revealed that types of lignin samples and solvent properties led to change in the lignin chemical linkage frequencies.

**Figure 1:**
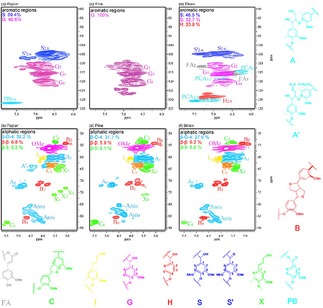
2D‐HSQC NMR spectra of the different types of lignin samples.^a^ (a) aromatic regions spectra analysis of the poplar lignin samples; (b) aliphatic regions spectra analysis of the poplar lignin samples; (c) aromatic regions spectra analysis of the pine lignin samples; (d) aliphatic regions spectra analysis of the pine lignin samples; (e) aromatic regions spectra analysis of the straw lignin samples; (f) aliphatic regions spectra analysis of the straw lignin samples. ^a^ Results expressed per 100 Ar based on quantitative 2D‐HSQC spectra; I (C_9_)=0.5I (S_2,6_)+I (G_2_)+0.5I (H_2,6_); β‐O‐4=I (β‐O‐4)/I (C_9_); β‐β=I (β‐β)/I (C_9_); β‐5=I (β‐5)/I (C_9_).11c

The internal linkages within different types lignin samples are confirmed by 2D‐HSQC NMR (Figure 1). The 2D‐HSQC NMR for identifying structures based on their chemical shifts.^11^ There are clear signals of syringyl (S), guaiacyl (G) and p‐hydroxyphenyl (H) units in the aromatic region (δC/δH 79–135/5.7–7.9 ppm). S units and G units are found as a highlighted signal in poplar lignin samples for the C_2,6_‐H_2,6_ at δC/δH 104.32/6.63 ppm, C_2_‐H_2_ at δC/δH 109.75/7.53 ppm, C_5_‐H_5_ at δC/δH 114.98/6.65 ppm and C_6_‐H_6_ at δC/δH 118.72/6.74 ppm, respectively. Pine lignin samples show structural differences notably. As shown in Figure 1c, the G structural units are the whole aromatic region of the pine lignin samples (100 wt %) and signals of S or H are not discovered, indicating that the pine lignin samples appertain to the G types. In Figure 1e, the amount of H, G and S units observed in the aromatic region are 20.8 wt %, 32.7 wt % and 46.5 wt %, respectively, suggesting straw lignin samples is a typical SGH types grass lignin.4b,11c

In the three types lignin samples aliphatic regions (δC/δH 49–91/2.4–5.8 ppm) of the 2D‐HSQC NMR (Figure 1), cross‐signals of methoxy groups (OMe), β‐O‐4 aryl ether linkages (A) and β‐5 linkages (C) were all recognizable. On the other hand, the corresponding anomeric correlations of β‐D‐xylopyranoside units (X_2_, X_3_, and X_4_) were tested for poplar lignin samples, demonstrating the existence of LCC structures in these lignin samples. This result indicates that poplar lignin samples LCC structures are not easier to dissolved in ethanol/water at 175 °C. Besides, a quantitative content analysis of internal linkages (β‐O‐4, β‐β and β‐5) and aromatic units was performed using the procedure described in, and the results are illustrated in Figure 1. The maximum content of β‐O‐4 bonds in the poplar lignin samples is 36.2/100 Ar. Meanwhile, the maximum content of β‐β linkages and β‐5 linkages in the poplar lignin samples is 6.8/100 Ar and 5.3/100 Ar (Figure 1b). It is reported that there are more C−O linkages (mostly β‐O‐4 aryl ether bonds) and C−C structure in lignin which are relatively more straightforward to depolymerize.^12^ Thus, the lignin such as the poplar lignin samples with higher β‐O‐4 content may be a better raw material for depolymerization, which provides a platform for the production of aromatic compounds.

### 2.2 GPC and Elemental Analysis of the Different Types of Lignin Samples

**Figure 2:**
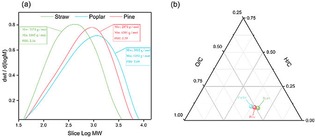
GPC and elemental analysis of the different types of lignin samples. (a) GPC of the different types of lignin samples; (b) elemental analysis of the different types of lignin samples.

GPC is performed to analyze the molecular weight distributions of the three types of lignin samples from ethanol pulping extraction. The results prove that the distribution curve of molecular weight exists to the high molecular weight area with all untreated primitive lignin. Meanwhile, samples contain both small and large molecular weight lignin fragments. In order to quantitatively compare the three types of lignin samples, Figure 2a illustrates the results of the weight‐average molecular weight (Mw), number‐average molecular weight (Mn) and polydispersity index (PDI). As expected, poplar has a relatively high molecular weight of 3912 g/mol (Mw) and a polydispersity of 2.89 compared with pine and straw. As compared to poplar and pine, straw lignin has less content of G (32.7 wt %) structure units and less content of H (20.8 wt %) structure units (2D‐HSQC NMR results in Figure 1), these units are randomly distributed in the straw lignin structure. This may result in its lower molecular weight.^13^ In parallel, straw lignin samples contain less β‐O‐4 aryl ether linkages, resulting in lower molecular weight.^13^ It is confirmed by analysis of the lignin fractions using elemental analysis and 2D‐HSQC NMR. Furthermore, the polydispersity of poplar lignin (2.89) is higher than that of pine lignin (2.39) and straw lignin (2.16), suggesting that the poplar lignin samples have broader molecular weight distributions. Figure 2b depicts the atomic ratios results of H/C and O/C for the three types of samples obtained after ethanol/water extraction at 175 °C. The O/C atomic ratio of the pine lignin samples and straw lignin samples are lower than that of the poplar lignin samples, whereas the H/C ratio remained at the same level. This result indicates that the poplar lignin samples may contain more oxygen functional groups, such as aryl−ether linkages, methoxyl, etc.

### 2.3 Yield of the Liquid Products, Solid Products and Bio‐Char

Upon effective extraction of the different types of lignin samples, these samples are applied to PTA catalysis in ethanol/water for the purpose of obtaining the aromatic compounds. After the catalysis, four products are isolated: phenolic monomers and three solid residues. The depolymerization of the three types of lignin samples with PTA catalyst is performed in ethanol/water under 250 °C for 6 h. The catalytic results for the three types of lignin samples conversion in ethanol/water are summarized in Table 1. We developed a comprehensive work‐up procedure to differentiate phenols, oligomers (ethanol‐soluble, EL), polymers (THF‐soluble, TL) and bio‐char.

**Table 1:** Mass yields of catalysis depolymerization products of the three types of lignin samples.

Lignin Types	Monomers (wt %)	EL‐ Soluble (wt %)	THF‐soluble (wt %)	Bio‐char (wt %)	Mass balance (wt %)
Poplar	8.06	53.53	31.49	2.06	95.14
Pine	5.44	52.62	32.44	2.53	93.03
Straw	4.52	51.64	32.67	3.96	92.79

The lignin types have a significant effect on the reaction yields. About the ethanol solvent lignin samples from different types under the same conditions, poplar lignin samples obtain the higher yields (8.06 wt %) of depolymerized mono phenols than pine lignin samples and straw lignin samples. The reason for this phenomenon may be that the PTA catalyst has an excellent selective activity in the depolymerization of the β‐O‐4 aryl ether bonds of three types of lignin samples. Simultaneously, Bouxin et al. discovered that the ration of β‐O‐4 linkages affects the yields and properties of phenolic monomers after depolymerization.^14^ In our research, the phenolic monomers yield of the three lignin samples are significantly different (Table 1, from 4.52 wt % to 8.06 wt %), which can easily to explain the effect of β‐O‐4 aryl ether bonds on the efficiency of phenolic monomers. Thus, based on the above study that PTA catalyst is an active effective catalyst for non‐noble metal and it accords good activity in catalyzing the β‐O‐4 bonds of biomass lignin.

### 2.4 Catalytic Conversion of the Model Compound and Lignin Samples

**Figure 3:**
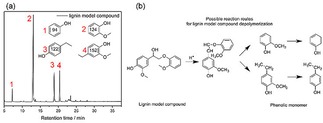
The reaction of the lignin model compound with the PTA catalyst.

To prove PTA has the potential as a catalyst for the depolymerization of β‐O‐4 linkages in the different types of lignin samples, lignin model dime compound is first used as substrates to perform the catalytic reaction. On treatment of lignin model dimer compound with PTA catalyst in ethanol/water during the same reaction temperature and time. The monomer products are extracted by ethyl acetate and analyzed by GC‐MS. In Figure 3a, no starting dimer is detected in the products of dimer catalyzed by PTA, indicating the lignin model dimer is converted completely. Four main products are found at the retention time of 7, 13, 19 and 21 min. Among these monomer products, phenol (94), 2‐methoxyphenol (124), 4‐ethylphenol (122) and 4‐ethyl‐2‐methoxyphenol (152) compounds both have representative meanings. The yields of four phenolic monomers are 30.22 wt %, 16.02 wt %, 14.38 wt %, and 8.61 wt %, respectively. The lignin model compound is 4‐(1‐hydroxy‐2‐(2‐methoxyphenoxy)ethyl)‐2‐methoxypheno. The reaction route of lignin model compound can be considered on the basis of the contents of earlier articles^15^ and is shown in Figure 3b. The reaction route of 2‐(4‐hydroxy‐3‐methoxyphenyl)‐acetaldehyde (152) and 2‐methoxyphenol (124) products are decisive for the main process. The abstraction of the β‐proton by a certain base to afford significantly acid labile enol ether compound, 1‐(2‐methoxyphenoxy)‐2‐(3,4‐dimethoxyphenyl) ethene, which cannot be detected under general acidolysis conditions. Then 1‐(2‐methoxyphenoxy)‐2‐(3,4‐dimethoxyphenyl) ethane is consecutively acid hydrolyzed at the β‐O‐4 bond to give 124 and 166. And the existence of this route is also confirmed in Yokoyama's article.^16^ In addition, large amounts of 94 and 120 are detected in the lignin model experiment, which indicates that not only lignin model compound is effectively catalyzed depolymerization by, PTA but also demethoxylation reaction has occurred between different structural units. These results indicate the complete cleavage of the β‐O‐4 bond in dimer after the catalytic depolymerization of the β‐O‐4 lignin model dimer by the PTA catalyst.

**Figure 4:**
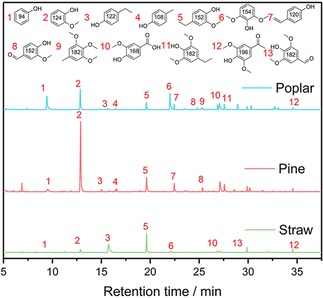
Gas chromatogram comparison of the three types of lignin samples under PTA catalyst.

In order to explore the effect of the different types of lignin samples during reactions of depolymerization. On treatment of the lignin samples with PTA catalyst in a mixture of ethanol/water in an autoclave, a mixture solution is obtained. After acidification, the products are extracted with ethyl acetate. The amount of the phenolic monomer is collected during the different reaction conditions, which are measured by GC‐MS and GC‐FID analyses, are detailed in Figure 4. Figure 4 exhibits mono phenolic compounds, including guaiacyl propanol, isoeugenol, guaiacyl propane, syringyl propene, syringyl propane and syringyl propanol, as well as 4‐ethyl‐phenol the main products, which are derived from the lignin p‐hydroxyphenyl, guaiacol, syringol, and their derivate. During the same reaction conditions, the species of lignin samples influence the types and content of each mono phenols compound. The inter‐unit aryl ether bond on the Cα or Cβ atom of the aliphatic side chain is depolymerized, resulting in a large quantity of aromatic compounds.^17^

**Figure 5:**
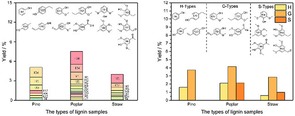
Yields distribution and types distribution of the phenolic monomers. (a) yields distribution of the phenolic monomers; (b) types distribution of the phenolic monomers.

Figure 5a summarizes the detail of the phenolic monomers yields from the different lignin samples. Regarding the lignin samples from various plants, straw lignin samples obtain the lower overall phenolic monomers yields (4.52 wt %) of depolymerized products than pine and poplar lignin samples (5.44 wt % and 8.06 wt %), which is in accordance with the fact that straw lignin samples possess less resistant linkages of β‐O‐4 (Figure 1). The results are also in consistent with Sels et. al. reported that lignin rich in S units and the more proportion of β‐O‐4 linkages are the ideal feedstock for the production of chemicals.^18^ And it can show higher depolymerization efficiency. This result is consistent with the yield sequence of mass and phenolic monomer (Table 1). In addition, it is well known that lignin depolymerization competes well with solid acid in catalytic process.^18^ So in here, two main products are also found at the retention time of 13 and 19 min which are 4‐ethylphenol and 4‐ethyl‐2‐methoxyphenol during all types of lignin conditions (Figure 4 and Figure 5). This indicates that this selective process of lignin is highly dependent on the PTA catalysts, and phenolic monomers basic units are formed by different types of lignin β‐O‐4 aryl ether bonds cleavage. The 4‐ethylphenol is a critical starting material for fine chemical industry and it can be readily converted to phenol and ethylene over acid aluminosilicates.^19^ And the 4‐ethyl‐2‐methoxyphenol also have a promising application for the pharmaceutical field and food industries.^20^ As abovementioned, the PTA catalyst is also the key to selective catalysis of lignin.

**Scheme 1:**
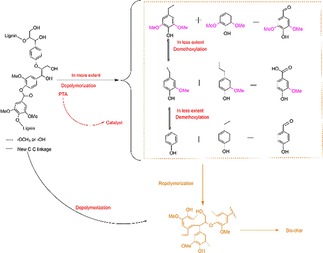
Possible reaction routes of lignin‐catalyzed depolymerization.

With respect to the quantified monomer ratios of the phenolic monomers, G and H units make up the primary monomers in the phenolic monomers of pine lignin samples, while G, H and S units are the dominant monomers in the phenolic monomers of poplar and straw lignin samples (Figure 5b). In Particular, the main component of the pine lignin samples is composed of G‐type units and H‐type units. The GC‐FID measurement provides a G : S : H percentage of 68 : 0 : 32, which is entirely different from the lignin structure. Similar results have shown in poplar lignin samples, S units content decrease from 59.4 wt % to 49.2 wt %, H units increase from 0 wt % to 8.3 wt % and G units increase from 40.6 wt % to 42.4 wt % in poplar phenolic monomers. From the above study, it means that the demethoxylation reaction occurs during the PTA catalytic depolymerization process. At the same time, it also discovers a large difference in the ratio of H, G and S structural signals between the phenolic monomers and the corresponding lignin samples. The conversion of the S‐type structural units and G‐type structural units during the PTA catalytic reaction are more favorable for the conversion of the H‐type structural units and G‐type structural units. These results fit well with lignin types (2D‐HSQC NMR results). In our catalytic depolymerization products, all phenolic monomers possible reaction route is shown in Scheme 1.

### 2.5 Characterization of Lignin Residue Products from Depolymerization

**Figure 6:**
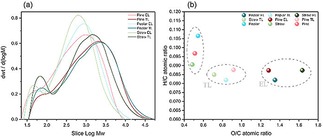
GPC and elemental analysis of the lignin residue products. (a) GPC of the lignin residue products; (b) elemental analysis of the lignin residue products.

Elemental and heterogeneity analyses of the residual lignin are essential to aid in understanding any depolymerization processes. The choice of solvent in the separation of lignin residual is important. According to Liu et al.^21^ the lignin depolymerization product was successfully separated into oligomers small molecule lignin residue and Huang et al.^22^ discovered a multimeric macromolecular lignin residue was isolated, so the ethanol and THF are selected as the solvent to separate lignin derivatives. The parts of the EL and TL with the yields from lignin depolymerization under the same reaction conditions are recorded (Table 1). The molecular weight of the catalysis products EL and TL are estimated by GPC and the changes of the different types of lignin residue samples products during the catalysis depolymerization of PTA catalyst are elucidated. The molecular weight distributions of the EL and TL after depolymerization reaction are shown in Figure 6a. It is found that the different types of lignin residue samples products (EL and TL) molecular weight is always less than untreated primitive lignin samples. Changes in the molecular weight of these EL and TL can affect the occurrence of lignin depolymerization and repolymerization during the reaction. And all of the molecular weight of the lignin residue products (EL and TL) with catalytic depolymerization are less than untreated primitive lignin samples. The phenomenon implies that the aryl ethers linkages of lignin could be depolymerized efficiently by PTA catalyst to form lignin fragments. In short, the effects of PTA catalyst accelerate lignin depolymerization. In addition, the lignin macro‐molecules are degraded into small molecules with a sharp drop in molecular weight. As the TL molecular weight content is higher than EL molecular weight, which means that the PTA catalytic depolymerization process can produce not only aromatic oligomers but also produce repolymerization reactions. Therefore, the EL molecular weight is always smaller than the TL molecular weight. These insights into the dominant reaction (depolymerization vs repolymerization) in the reaction process is important for a further study of avoiding lignin repolymerization during the depolymerization process.

Figure 6b shows the H/C and O/C of the EL and TL lignin samples obtained after catalytic depolymerization. Compare to the untreated primitive lignin samples, both the O/C of lignin residue samples EL and TL are increased and the H/C of lignin residue samples EL and TL are decreased after ethanol/water depolymerization at 250 °C. The sharp increase in the O/C ratio content is due to the depolymerization reaction occurring under the PTA catalytic reaction to breaking a large amount of side chain alkane functional methyl, ethyl and vinyl. In addition, the O/C ratio content of all lignin residue EL is larger than TL because a large amount of condensation reaction occurs in the TL, and a new C−C bond can be formed. This result shows that the repolymerization and depolymerization of lignin fragments followed by intramolecular demethoxylation are always present throughout all the depolymerization time. These are in agreement with the GPC and 2D‐HSQC NMR analysis.

**Figure 7:**
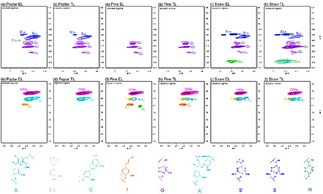
2D‐HSQC NMR spectra of the lignin residue products. (a) aromatic regions spectra analysis of the poplar EL residue products; (b) aliphatic regions spectra analysis of the poplar EL residue products; (c) aromatic regions spectra analysis of the poplar TL residue products; (d) aliphatic regions spectra analysis of the poplar TL residue products; (e) aromatic regions spectra analysis of the pine EL residue products; (f) aliphatic regions spectra analysis of the pine EL residue products; (g) aromatic regions spectra analysis of the pine TL residue products; (h) aliphatic regions spectra analysis of the pine TL residue products; (i) aromatic regions spectra analysis of the straw EL residue products; (j) aliphatic regions spectra analysis of the straw EL residue products; (k) aromatic regions spectra analysis of the straw TL residue products; (l) aliphatic regions spectra analysis of the straw TL residue products.

In order to reveal the changes in the lignin samples after catalytic depolymerization, all residual lignin samples are analyzed by 2D‐HSQC NMR methodology. It is clear that most of A (β‐O‐4), B (β‐β) and C (β‐5) linkages disappear in the side chain regions after PTA catalyst, implying extensive depolymerization removal of the β‐O‐4 aryl ether bonds (Figure 7). Besides, it has been found that both of the β‐ether and α‐ether bonds of β‐O‐4 and α‐O‐4 linkages in lignin are easily depolymerization in this system, while the 5–5 type and the aromatic ring structure are correspondingly steady. In the presence of PTA, lignin cannot be fully degraded into oligomers and monomers during depolymerization, accompanied by condensation reactions. These studies support the GPC results discussed above (Figure 6a) that the majority of the lignin depolymerization products are phenolic monomers and small molecule lignin. In the aromatic region, the area of H, S and G signals of the lignin residues samples change significantly before and after the reaction. This indicates that most of the aromatic rings remain in the depolymerization process, and it can be concluded that this mild but effective PTA catalytic system is not only active for the selective oxidative depolymerization of β‐O‐4 aliphatic C−C and C−O bonds but also it is suitable for different types of lignin depolymerization to form valuable aromatic compounds. Furthermore, it can be seen that with separation of lignin residues, the normal G and S signals of the lignin residues samples are gradually reduced, especially the signal of G_6_, while the signal of condensed G and S signals (TL) increased, indicating the condensation of the TL is high.

## 3 Conclusions

In summary, PTA catalytic depolymerization approach for lignin provided a promising strategy to produce phenolic monomers from depolymerization of native lignin. The present work also showed that the yields and distribution of phenolic monomers products depended strongly on the lignin structures. The model compound study confirmed that improved phenolic monomers were obtained when lignin model compounds were used over PTA polyoxometallate catalysts. Straw lignin samples showed lower depolymerizing efficiency than poplar lignin samples, mainly due to the existence of less aryl ether linkages of β‐O‐4 Different types of lignin samples showed different H/G/S ratios after PTA catalysis. Comparison between lignin and depolymerization products by elemental analysis, GPC, 2D‐HSQC NMR and GC‐MS analysis suggested that PTA exhibited high activity in β‐O‐4 cleavage and the conversion of different types of lignin by PTA catalyst provided us a highly selective strategy. We believe the work described here was of real importance to change the distribution of products types, thereby greatly increasing the selectivity of phenolic monomers.

## Experimental Section

**Materials**. All the poplar, pine and straw were purchased from Jining Ming Sheng New Material Co., Ltd., Jinan, China. All commercial reagents were directly used without any purification. The catalyst phosphotungstic acid (PTA), dimethyl sulfoxide (DMSO), tetrahydrofuran (THF) and ethanol were supplied by Sinopharm Chemical Reagent Co. Ltd. Standard samples of N‐tetradecane, hydrochloric acid (HCl), and ethyl acetate were obtained from Aladdin Chemical Reagent Co. Ltd. The lignin model compound was purchased from a local factory.

**Preparation of the Lignin Samples**. Three types of lignin samples were extracted using in this study. Eighty grams (80 g) of samples was treated with ethanol/water (v/v, 60 wt %) mixture in 175 °C for 100 min. The mixture was cooled, filtered, and washed with solvent ethanol. The liquid part was treated to precipitate lignin: liquid was poured into acid water. Then separated by filtration and freeze‐dried to obtain a dark brown solid.

**Depolymerization of the Lignin Model Compound**. Catalytic depolymerization experiments were conducted in 50 mL Parr reactor equipped. Typically, the lignin model compound (0.05 g), PTA catalyst (0.05 g), H_2_O_2_ (0.2 ml) and ethanol/water (6 mL) were combined. The mixture was stirred in the reactor at 250 °C for 6 h. After the reaction, ethanol was evaporated and deionized/water was added, and the reaction solution was adjusted to pH=2.0. Finally, products were extracted using ethyl acetate.

**Scheme 2:**
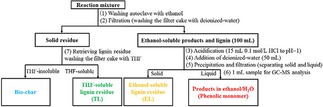
Work‐up procedure of reaction product mixture.

**Depolymerization of the Lignin Samples**. Catalytic depolymerization experiments were also conducted in a 100 mL Parr reactor equipped. Typically, lignin (0.5 g), PTA catalyst (0.25 g), H_2_O_2_ (2 ml) and ethanol/water (60 mL) were combined. The reaction condition was 250 °C for 6 h under stirring. After the reaction, the mixture quenched in the water. Yellow and black solids were observed at the bottom of the container. A workup procedure was developed to distinguish the liquid products from the solid residues as shown in Scheme 2.

**Characterization of the Product Samples**. Before the test, all lignin samples were dried at 40 °C in vacuum. All samples were used without further treatment. The molecular weights of lignin preparations were analyzed by gel permeation chromatography (GPC).11b Analyses were carried out at 25 °C using THF as an eluent with a flow rate of 1 ml/min. For the lignin sample analysis, the sample was prepared at a concentration of 10 mg/ml. All samples were filtered using a 0.45 μm filter membrane prior to injection. The contents of C, H, N and S were analyzed by Vario EL III elemental analyzer (Hanau, Germany). The 2D‐HSQC NMR determinations for all prepared lignin samples were carried out in a Bruker AVIII 400 MHz spectrometer. The 2D‐HSQC NMR determination was carried out according to a recently reported method.17b,^23^ The spectral widths for the 2D‐HSQC NMR were 20000 Hz and 5000 Hz, respectively. We analyzed and assigned 2D‐HSQC NMR cross signals compared with previous literature.11a,^23^ Gas chromatography spectrometry (GC‐MS, Agilent 5890) was used to identify the components of volatile product, with a column of HP‐5 (30 m×0.25 μm×0.25 μm). The injector was maintained at 280 °C in split mode with helium carrier gas. The GC was used for quantitative analysis, with a flame ionization detector (FID) and an HP‐5 column. The temperature program was the same as the GC‐MS analysis. All the quantitative analyses of liquid phase products were based on 1 mL GC‐FID with n‐tetradecane as an internal standard. The FID response factors were calculated using the active carbon number (ECN) method to determine the relative response factors, which were corrected by the molecular weight of the compounds relative to N‐tetradecane.^23^ The yields of phenolic monomers, lignin residues and char yields (in wt %) were calculated according to the following equations (1)–(4):

Yield of phenolic monomers (wt %):

(1) $$=\frac{wt.\;of\;monomers\;(calc.\;from\;GC-FID)}{wt.\;of\;untreated\;lignin}\times 100\;\%$$

Yield of ethanol‐soluble lignin (EL, wt %):

(2) $$=\frac{wt.\;of\;EL}{wt.of\;untreated\;lignin}\times 100\;\%$$

Yield of tetrahydrofuran‐soluble lignin (TL, wt %):

(3) $$=\frac{wt.\;of\;TL}{wt.\;of\;untreated\;lignin}\times 100\;\%$$

Yield of bio‐char (wt %):

(4) $$=\frac{wt.\;of\;bio-char}{wt.\;of\;untreated\;lignin}\times 100\;\%$$

## Conflict of interest

The authors declare no conflict of interest.

## References

[1] J. Zakzeski , P. C. Bruijnincx , A. L. Jongerius , B. M. Weckhuysen , Chem. Rev. 2010, 110, 3552–3599. 20218547 10.1021/cr900354u

[2a] D. Kai , M. J. Tan , P. L. Chee , Y. K. Chua , Y. L. Yap , X. J. Loh , Green Chem. 2016, 18, 1175–1200;

[2b] C. Liu , J. Hu , H. Zhang , R. Xiao , Fuel. 2016, 182, 864–870.

[3] P. Azadi , O. R. Inderwildi , R. Farnood , D. A. King , Renewable Sustainable Energy Rev. 2013, 21, 506–523.

[4a] C. Li , X. Zhao , A. Wang , G. W. Huber , T. Zhang , Chem. Rev. 2015, 115, 11559–11624; 26479313 10.1021/acs.chemrev.5b00155

[4b] H. Guo , D. M. Miles-Barrett , B. Zhang , A. Wang , T. Zhang , N. J. Westwood , C. Li , Green Chem. 2019, 21, 803–811.

[5] F. G. Calvo-Flores , J. A. Dobado , ChemSusChem. 2010, 3, 1227–1235. 20839280 10.1002/cssc.201000157

[6a] Y.-C. Sun , M. Wang , R.-C. Sun , ACS Sustainable Chem. Eng. 2015, 3, 2443–2451;

[6b] T. Q. Yuan , S. N. Sun , F. Xu , R. C. Sun , J. Agric. Food Chem. 2011, 59, 6605–6615. 21568341 10.1021/jf2003865

[7] H. Guo , B. Zhang , C. Li , C. Peng , T. Dai , H. Xie , A. Wang , T. Zhang , ChemSusChem. 2016, 9, 3220–3229. 27791336 10.1002/cssc.201600901

[8] H. Yang , K. Norinaga , J. Li , W. Zhu , H. Wang , Fuel Process. Technol. 2018, 181, 207–214.

[9] H. Deng , L. Lin , Y. Sun , C. Pang , J. Zhuang , P. Ouyang , J. Li , S. Liu , Energy Fuels. 2008, 23, 19–24.

[10] Z. Du , J. Ma , F. Wang , J. Liu , J. Xu , Green Chem. 2011, 13.

[11a] X. Wang , Y. Guo , J. Zhou , G. Sun , RSC Adv. 2017, 7, 8314–8322;

[11b] C. Liu , X. Wang , F. Lin , H. Zhang , R. Xiao , Fuel Process. Technol. 2018, 169, 50–57;

[11c] B. Wang , X.-J. Shen , J.-L. Wen , R.-C. Sun , RSC Adv. 2016, 6, 57986–57995.

[12a] X. Huang , O. M. M. Gonzalez , J. Zhu , T. I. Korányi , M. D. Boot , E. J. Hensen , Green Chem. 2017, 19, 175–187;

[12b] C. Liu , S. Wu , H. Zhang , R. Xiao , Fuel Process. Technol. 2019, 191, 181–201.

[13] H. Guo , B. Zhang , Z. Qi , C. Li , J. Ji , T. Dai , A. Wang , T. Zhang , ChemSusChem. 2017, 10, 523–532. 27863130 10.1002/cssc.201601326

[14] F. P. Bouxin , A. McVeigh , F. Tran , N. J. Westwood , M. C. Jarvis , S. D. Jackson , Green Chem. 2015, 17, 1235–1242.

[15a] T. Imai , T. Yokoyama , Y. Matsumoto , J. Wood Sci. 2011, 57, 219–225;

[15b] H. Ito , T. Imai , K. Lundquist , T. Yokoyama , Y. Matsumoto , J. Wood Chem. Technol. 2011, 31, 172–182.

[16] T. Yokoyama , J. Wood Chem. Technol. 2014, 35, 27–42.

[17a] Z. Li , J. Cao , K. Huang , Y. Hong , C. Li , X. Zhou , N. Xie , F. Lai , F. Shen , C. Chen , Bioresour. Technol. 2015, 177, 159–168; 25485736 10.1016/j.biortech.2014.11.043

[17b] C. Peng , Q. Chen , H. Guo , G. Hu , C. Li , J. Wen , H. Wang , T. Zhang , Z. K. Zhao , R. Sun , H. Xie , ChemCatChem. 2017, 9, 1135–1143;

[17c] S. Van den Bosch , W. Schutyser , R. Vanholme , T. Driessen , S. F. Koelewijn , T. Renders , B. De Meester , W. J. J. Huijgen , W. Dehaen , C. M. Courtin , B. Lagrain , W. Boerjan , B. F. Sels , Energy Environ. Sci. 2015, 8, 1748–1763.

[18] W. Schutyser , T. Renders , S. Van den Bosch , S. F. Koelewijn , G. T. Beckham , B. F. Sels , Chem. Soc. Rev. 2018, 47, 852–908. 29318245 10.1039/c7cs00566k

[19] Y. Liao , M. d'Halluin , E. Makshina , D. Verboekend , B. F. Sels , Appl. Catal. B 2018, 234, 117–129.

[20a] D. R. Vardon , M. A. Franden , C. W. Johnson , E. M. Karp , M. T. Guarnieri , J. G. Linger , M. J. Salm , T. J. Strathmann , G. T. Beckham , Energy Environ. Sci. 2015, 8, 617–628;

[20b] S. Y. Lee , S. H. Hong , S. H. Lee , S. J. Park , Macromol. Biosci. 2004, 4, 157–164. 15468205 10.1002/mabi.200300096

[21] Q. Liu , P. Li , N. Liu , D. Shen , Polym. Degrad. Stab. 2017, 135, 54–60.

[22] X. Huang , T. I. Koranyi , M. D. Boot , E. J. Hensen , ChemSusChem. 2014, 7, 2276–2288. 24867490 10.1002/cssc.201402094

[23] X. Wang , B. Du , L. Pu , Y. Guo , H. Li , J. Zhou , J. Anal. Appl. Pyrolysis. 2018, 129, 13–20.

